# Comparison of clinical outcomes and left ventricular remodeling after ST-elevation myocardial infarction between patients with and without diabetes mellitus

**DOI:** 10.1007/s00380-021-01827-w

**Published:** 2021-03-14

**Authors:** Naoyuki Akashi, Takunori Tsukui, Kei Yamamoto, Masaru Seguchi, Yousuke Taniguchi, Kenichi Sakakura, Hiroshi Wada, Shin-ichi Momomura, Hideo Fujita

**Affiliations:** Division of Cardiovascular Medicine, Saitama Medical Center, Jichi Medical University, 1-847 Amanuma, Omiya-ku, Saitama, 330-8503 Japan

**Keywords:** ST-elevation myocardial infarction, Diabetes mellitus, Left ventricular remodeling, Heart failure hospitalization

## Abstract

Left ventricular remodeling (LVR) after ST-elevation myocardial infarction (STEMI) is generally thought to be an adaptive but compromising phenomenon particularly in patients with diabetes mellitus (DM). However, whether the extent of LVR is associated with poor prognostic outcome with or without DM after STEMI in the modern era of reperfusion therapy has not been elucidated. This was a single-center retrospective observational study. Altogether, 243 patients who were diagnosed as having STEMI between January 2016 and March 2019, and examined with echocardiography at baseline (at the time of index admission) and mid-term (from 6 to 11 months after index admission) follow-up were included and divided into the DM (*n* = 98) and non-DM groups (*n* = 145). The primary outcome was major adverse cardiovascular events (MACEs) defined as the composite of all-cause death, heart failure (HF) hospitalization, and non-fatal myocardial infarction. The median follow-up duration was 621 days (interquartile range: 304–963 days). The DM group was significantly increased the rate of MACEs (*P* = 0.020) and HF hospitalization (*P* = 0.037) compared with the non-DM group, despite of less LVR. Multivariate Cox regression analyses revealed that the patients with DM after STEMI were significantly associated with MACEs (Hazard ratio [HR] 2.79, 95% confidence interval [CI] 1.20–6.47, *P* = 0.017) and HF hospitalization (HR 3.62, 95% CI 1.19–11.02, *P* = 0.023) after controlling known clinical risk factors. LVR were also significantly associated with MACEs (HR 2.44, 95% CI 1.03–5.78, *P* = 0.044) and HF hospitalization (HR 3.76, 95% CI 1.15–12.32, *P* = 0.029). The patients with both DM and LVR had worse clinical outcomes including MACEs and HF hospitalization, suggesting that it is particularly critical to minimize LVR after STEMI in patients with DM.

## Introduction

Among patients with acute myocardial infarction (AMI), left ventricular remodeling (LVR) is thought to occur as an adaptive phenomenon that later results in structural and functional changes such as left ventricular dilatation and reduction of ejection fraction (LVEF) in response to myocardial injury [1–3]. According to the historical definition of > 15% increase in left ventricular end-systolic volume (LVESV), LVR is observed in as many as 30% of anterior myocardial infarction (MI) cases and approximately 17% of non-anterior MI cases even with timely primary coronary intervention (PCI) and the use of cardiovascular-protective drugs such as angiotensin-converting enzyme inhibitors or angiotensin II receptor blockers, beta blockers, mineral corticoid receptor antagonists, and statins [4]. Diabetes alone is known to cause LVR [5, 6], and in another concept of diabetes mellitus-related cardiomyopathy (DMCMP), LVR has no other cause besides DM [7].

The extent of LVR is generally believed to be associated with worse long-term clinical outcome along with progression of heart failure (HF). However, whether concomitant diabetes mellitus (DM) is associated with a greater extent of LVR that leads to worse clinical outcome in patients with ST-elevation myocardial infarction (STEMI) is still not precisely understood. The purpose of the present study was to elucidate the association between LVR and DM in patients with STEMI who underwent a successful modern acute reperfusion therapy mainly with primary PCI followed by optimal medical therapy (OMT).

## Materials and methods

### Study design and population

A single-center retrospective observational study was conducted. We identified patients with AMI from hospital records in our medical center from January 2016 to March 2019. We included patients with STEMI who underwent an echocardiographic examination at the time of index admission (baseline) and mid-term (from 6 to 11 months) follow-up. The patients were divided into the DM group (patients with diabetes mellitus on admission) and non-DM group (patients without diabetes mellitus on admission).

### Data collection, endpoints and definitions

Echocardiography was performed by experienced ultrasonographers. Clinical characteristics and outcomes were compared between the DM and non-DM groups. The primary outcomes included major cardiovascular events (MACEs) defined as the composite of all-cause death, HF hospitalization, and non-fatal MI. The secondary outcomes included differences in LVEF, left ventricular mass index (LVMI), left atrial volume index (LAVI), relative wall thickness (RWT), left ventricular end-diastolic volume index (LVEDVI), left ventricular end-systolic volume index (LVESVI), and brain natriuretic peptide (BNP) levels between the index admission and mid-term follow-up. This study was approved by the institutional review board, and written informed consent was waived because of the retrospective study design.

The diagnosis of AMI requires meeting the following criteria: symptoms consistent with AMI; elevated cardiac markers, including cardiac troponin T, troponin I, and/or creatinine phosphokinase (CK; at least twofold increase from the normal upper limit); and ST-segment elevation or depression on electrocardiography compatible with AMI [8–10]. Diagnostic ST-segment elevation was defined as a new ST-segment elevation at the J point in at least 2 contiguous leads of 2 mm (0.2 mV), and others were defined as not an ST-segment elevation [11]. Hypertension was defined as a systolic blood pressure (SBP) of ≥ 140 mmHg, diastolic blood pressure (DBP) of ≥ 90 mmHg, or a medical treatment for hypertension before admission [12]. Dyslipidemia was defined as a total cholesterol level of ≥ 220 mg/dl or a low-density lipoprotein (LDL) cholesterol level of ≥ 140 mg/dl or medical treatment for dyslipidemia [13]. DM was defined as a hemoglobin A1C level of ≥ 6.5% (as the national glycohemoglobin standardization program value), medical treatment for DM, or a history of DM [13]. Stress hyperglycemia, or a predictor of survival and increased risk of adverse events in patients both with and without DM, was defined as admission blood plasma glucose > 140 mg/dl both with and without a history of DM [14]. Shock was defined as a SBP of < 90 mmHg, use of vasopressors to maintain blood pressure, or an attempt of cardiopulmonary resuscitation [15, 16]. We calculated the estimated glomerular filtration rate (eGFR) from the serum creatinine level at admission, age, weight, and sex, using the following formula: eGFR = 194 × Cr^1.094^ × age^0.287^ (male), eGFR = 194 × Cr^1.094^ × age^0.287^ × 0.739 (female) [17]. Dual antiplatelet therapy was defined as a combination of antiplatelet medications such as aspirin, clopidogrel, prasugrel, and ticlopidine. We calculated LVMI from interventricular septum thickness (IVST), left ventricular internal dimension in diastole (LVDd), and posterior left ventricular wall thickness (PWT) using the formula recommended by the American Society of Echocardiography as follows: ${\text{LVMI}}\, = \,\left\{ {0.{8}\, \times \,{1}.0{4}\, \times \,\left[ {\left( {{\text{IVST}}\, + \,{\text{LVDd}}\, + \,{\text{PWT}}} \right)^{{3}} \, - \,{\text{LVDd}}^{{3}} } \right]\, + \,0.{6}} \right\}/{\text{body surface area}}$ [18]. RWT was calculated as follows: ${\text{RWT}}\, = \,{2}\, \times \,{\text{PWT}}/{\text{LVDd}}$ [18]. LVEF, LVEDVI, LVESVI, and LAVI were calculated using the modified Simpson’s method from two-dimensional, apical, two-chamber, and four-chamber views [18]. Remodelers were defined as the patients with LVR, which is an increase in left ventricular end-diastolic volume (LVEDV) between baseline and mid-term follow-up as a continuous variable [19]. Primary PCI was performed within 24 h of onset using standard techniques via the radial, femoral, or brachial artery. First, we advanced a conventional guidewire across the lesion and used a small balloon or thrombus aspiration catheter. The choice of devices was left to the discretion of each interventional cardiologist. The activated coagulation time was maintained > 250 s during PCI.

### Statistical analysis

Data are shown as percentage for categorical variables or mean ± SD for continuous variables. Categorical variables are presented as numbers (percentage) and compared using the chi-square or Fisher’s exact test. The Shapiro–Wilk test was performed to determine if the continuous variables were normally distributed. Normally distributed continuous variables were compared between the groups using an unpaired Student *t* test. Otherwise, continuous variables were compared using the Mann–Whitney *U* test. Event-free survival curves for MACEs were constructed with the Kaplan–Meier method, and the statistical differences between the curves were assessed using the log-rank test. Multivariate Cox regression analysis was performed to find the determinant of MACEs or HF hospitalization. Selected variables including age [20], sex [21], LVR [22] and parameters of glucose metabolism (model 1: DM [23], model 2: stress hyperglycemia [14]) were adopted as independent variables. The hazard ratio (HR) and the 95% confidence interval (CI) were calculated. All statistical tests were two-sided, and a *P* value of < 0.05 was considered statistically significant. We analyzed all data using SPSS ver. 25 for Windows (SPSS, Inc., Chicago, Illinois).

## Results

**Fig. 1:**
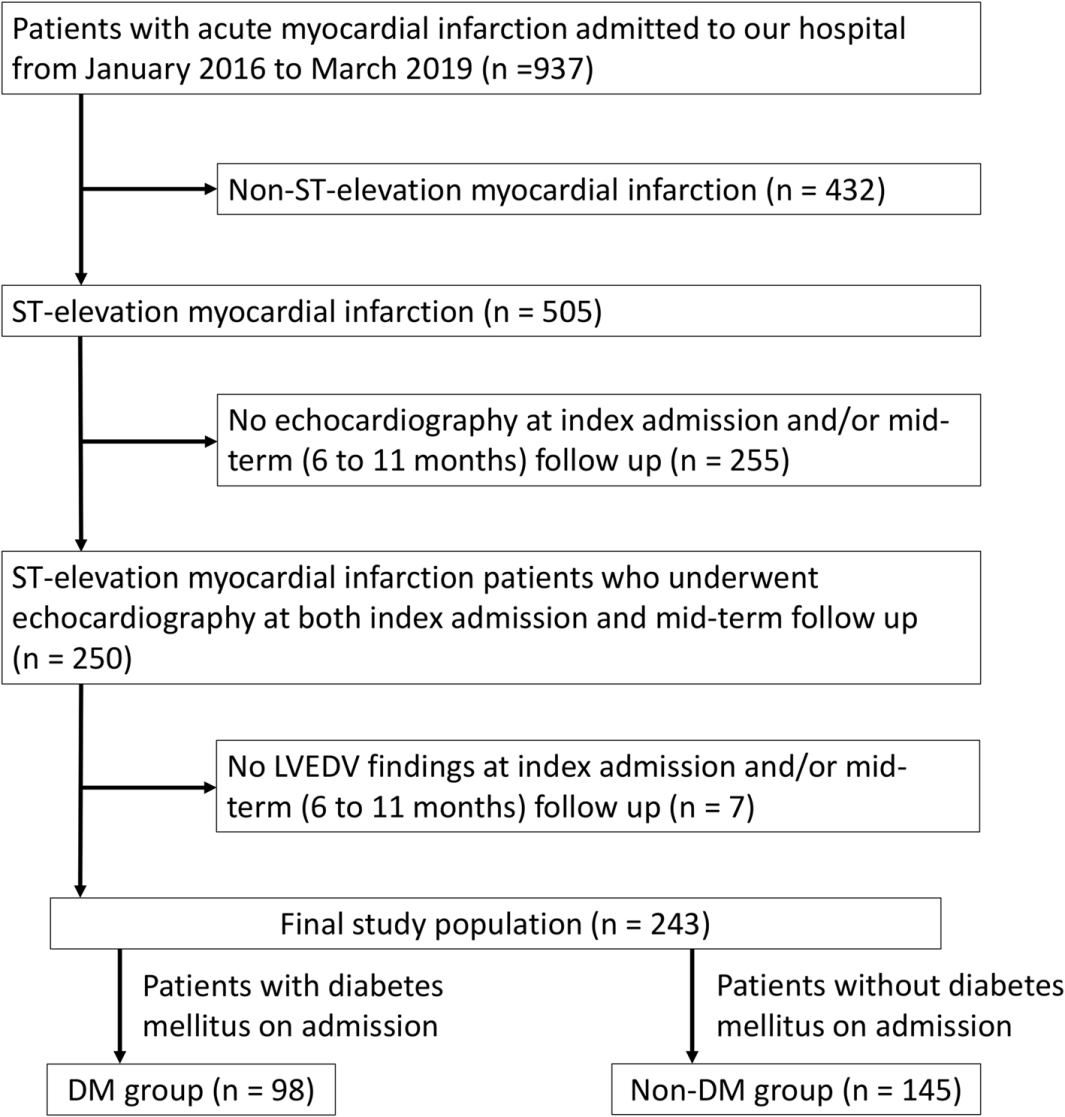
Study flow chart. *LVEDV* left ventricular end-diastolic volume, *DM* diabetes mellitus

Altogether, 937 patients with AMI were admitted to our medical center from January 2016 to March 2019. We excluded patients with non-STEMI (*n* = 432), patients with STEMI who did not undergo echocardiography at index admission and/or mid-term follow-up (*n* = 255), and patients who did not exist LVEDV findings at index admission and/or mid-term follow-up (*n* = 7). The final study population included 243 patients, who were divided into the DM (*n* = 98) and non-DM groups (*n* = 145). The study flowchart is shown in Fig. 1.

The comparison of patient characteristics between the 2 groups is shown in Table 1. The proportion of patients with a history of previous PCI was significantly higher in the DM group (13.3%) than in the non-DM group (4.8%; *P* = 0.019). The peak CK level was significantly lower in the DM group (1660.5 ± 1491.3 IU/L) than in the non-DM group (2731.6 ± 2666.4 IU/L; *P* = 0.002). Table 2 shows the angiographic characteristics between the 2 groups. Although the difference was not significant, the patients in the DM group tended to have more diseased vessels than those in the non-DM group (*P* = 0.081).

**Table 1:** Patient characteristics between the DM and the non-DM groups

	All (*n* = 243)	DM group (*n* = 98)	non-DM group (n = 145)	*P* value
Age, year (*n*, %)	67.0 ± 13.1 (243/243, 100)	66.7 ± 12.0	67.2 ± 13.9	0.74
Female sex, *n* (%)	47/243 (19.3)	14/98 (14.3)	33/145 (22.8)	0.10
Height, cm (*n*, %)	163.6 ± 9.2 (243/243, 100)	164.0 ± 9.0	163.3 ± 9.3	0.64
Weight, kg (*n*, %)	65.1 ± 12.3 (243/243, 100)	67.0 ± 12.8	63.9 ± 11.9	0.058
Body mass index, kg/m2 (*n*, %)	24.2 ± 3.3 (243/243, 100)	24.8 ± 3.5	23.8 ± 3.1	0.019
Hypertension, *n* (%)	173/243 (71.2)	75/98 (76.5)	98/145 (67.6)	0.13
Dyslipidemia, *n* (%)	129/243 (53.1)	54/98 (55.1)	75/145 (51.7)	0.61
Diabetes mellitus, *n* (%)	98/243 (40.3)			
Stress hyperglycemia, *n* (%)	143/243 (58.8)	85/98 (86.7)	58/145 (40.0)	< 0.001
Current smoker, *n* (%)	89/243 (36.6)	36/98 (36.7)	53/145 (36.6)	0.98
Past smoker, *n* (%)	88/243 (36.2)	37/98 (37.8)	51/145 (35.2)	0.68
History of previous CABG, n (%)	1/243 (0.4)	0/98 (0)	1/145 (0.7)	1.00
History of previous PCI, *n* (%)	20/243 (8.2)	13/98 (13.3)	7/145 (4.8)	0.019
History of previous MI, *n* (%)	15/243 (6.2)	9/98 (9.2)	6/145 (4.1)	0.11
History of previous HF, *n* (%)	1/243 (0.4)	1/98 (1.0)	0/145 (0)	0.40
*Killip classification*				0.89
1 or 2, *n* (%)	219/243 (90.1)	88/98 (89.8)	131/145 (90.3)	
3 or 4, *n* (%)	24/243 (9.9)	10/98 (10.2)	14/145 (9.7)	
Systolic blood pressure on admission, mm Hg (*n*, %)	137.1 ± 29.2 (240/243, 98.8)	142.0 ± 30.0	133.9 ± 28.3	0.041
Diastolic blood pressure on admission, mm Hg (*n*, %)	82.0 ± 18.4 (240/243, 98.8)	80.5 ± 18.2	83.0 ± 18.6	0.53
Shock on admission, *n* (%)	20/243 (8.2)	8/98 (8.2)	12/145 (8.3)	0.98
Cardiopulmonary arrest at out of hospital or admission, *n* (%)	8/243 (3.3)	2/98 (2.0)	6/145 (4.1)	0.48
Door to balloon time, min (n, %)	75.0 ± 37.3 (213/243, 87.7)	81.9 ± 44.4	70.7 ± 31.4	0.12
*Laboratory data*				
Hemoglobin A1C, % (*n*, %)	6.6 ± 1.5 (240/243, 98.8)	7.9 ± 1.7	5.8 ± 0.32	< 0.001
Admission blood plasma glucose, mg/dl (*n*, %)	173.6 ± 79.3 (240/243, 98.8)	222.2 ± 92.3	141.8 ± 48.1	< 0.001
Total cholesterol, mg/dl (*n*, %)	189.8 ± 41.5 (238/243, 97.9)	184.6 ± 42.4	193.2 ± 40.7	0.065
LDL cholesterol, mg/dl (*n*, %)	114.0 ± 37.4 (241/243, 99.2)	106.2 ± 36.5	119.3 ± 37.2	0.004
HDL cholesterol, mg/dl (*n*, %)	45.4 ± 11.3 (240/243, 98.8)	44.5 ± 12.4	46.0 ± 10.5	0.15
Triglyceride, mg/dl (*n*, %)	126.5 ± 82.8 (243/243, 100)	138.4 ± 97.8	118.5 ± 70.1	0.33
Creatinine, mg/dl (*n*, %)	1.1 ± 1.4 (243/243, 100)	1.5 ± 2.0	0.85 ± 0.33	0.16
eGFR, ml/min/1.73m^2^ (*n*, %)	70.4 ± 27.8 (243/243, 100)	66.4 ± 32.1	73.1 ± 24.3	0.082
C-reactive protein, mg/dl (*n*, %)	1.5 ± 3.5 (243/243, 100)	1.9 ± 3.7	1.3 ± 3.4	0.031
Uric acid, mg/dl (*n*, %)	5.7 ± 1.4 (242/243, 99.6)	5.4 ± 1.5	5.8 ± 1.4	0.012
BNP, pg/ml (*n*, %)	239.3 ± 432.8 (236/243, 97.1)	319.6 ± 486.5	186.1 ± 385.8	0.005
Peak CK, IU/L (*n*, %)	2299.6 ± 2323.6 (243/243, 100)	1660.5 ± 1491.3	2731.6 ± 2666.4	0.002
Peak CK-MB, IU/L (*n*, %)	214.7 ± 216.9 (243/243, 100)	150.1 ± 153.5	258.3 ± 241.7	< 0.001
*Medication on discharge*				
*Antiplatelet agents*				
Dual antiplatelet therapy, *n* (%)	229/243 (94.2)	94/98 (95.9)	135/145 (93.1)	0.36
*Antihypertensive medications*				
ACE-Is, *n* (%)	210/243 (86.4)	80/98 (81.6)	130/145 (89.7)	0.073
ARBs, *n* (%)	28/243 (11.5)	16/98 (16.3)	12/145 (8.3)	0.054
ACE-Is or ARBs, *n* (%)	235/243 (96.7)	94/98 (95.9)	141/145 (97.2)	0.57
β-blockers, *n* (%)	236/243 (97.1)	95/98 (96.9)	141/145 (97.2)	1.00
Mineralocorticoid receptor antagonists, *n* (%)	38/243 (15.6)	15/98 (15.3)	23/145 (15.9)	0.91
Loop diuretics, *n* (%)	52/243 (21.4)	22/98 (22.4)	30/145 (20.7)	0.74
Thiazide, *n* (%)	2/243 (0.8)	2/98 (2.0)	0/145 (0)	0.16
Tolvaptan, *n* (%)	6/243 (2.5)	4/98 (4.1)	2/145 (1.4)	0.22
Nicorandil, *n* (%)	1/243 (0.4)	0/98 (0)	1/145 (0.7)	1.0
Calcium channel blockers, *n* (%)	24/243 (9.9)	13/98 (13.3)	11/145 (7.6)	0.15
Statin, *n* (%)	239/243 (98.4)	96/98 (98.0)	143/145 (98.6)	1.00
*Antidiabetic medications*				
SGLT2 inhibitors, *n* (%)	23/243 (9.5)	23/98 (23.5)		
DPP-4 inhibitors, *n* (%)	66/243 (27.2)	66/98 (67.3)		
GLP-1 receptor agonists, *n* (%)	2/243 (0.8)	2/98 (2.0)		
Metformin, *n* (%)	17/243 (7.0)	17/98 (17.3)		
Sulfonylurea, *n* (%)	13/243 (5.3)	13/98 (13.3)		
Thiazolidine, *n* (%)	2/243 (0.8)	2/98 (2.0)		
Glinide, *n* (%)	4/243 (1.6)	4/98 (4.1)		
α-glucosidase inhibitors, *n* (%)	5/243 (2.1)	5/98 (5.1)		
Insulin, *n* (%)	16/243 (6.6)	16/98 (16.3)		
No medications, *n* (%)	16/243 (6.6)	16/98 (16.3)		

*CABG* coronary artery bypass grafting, *PCI* percutaneous coronary intervention, *MI* myocardial infarction, *HF* heart failure, *HDL* high-density lipoprotein, *LDL* low-density lipoprotein, *eGFR* estimated glomerular filtration rate, *BNP* brain natriuretic peptide, *CK* creatine kinase, *ACE-Is* angiotensin converting enzyme-inhibitors, *ARBs* angiotensin receptor blockers, *SGLT* sodium glucose transporter, *DPP* dipeptidyl peptidase, *GLP* glucagon like peptide

**Table 2:** Angiographic characteristics between the DM and the non-DM groups

	All (*n* = 243)	DM group (*n* = 98)	non-DM group (*n* = 145)	*P* value
Primary PCI, *n* (%)	196/243 (80.7)	75/98 (76.5)	121/145 (83.4)	0.18
*Culprit of AMI, n (%)*				1.00
Left main trunk and/or Left anterior descending artery	137/243 (56.4)	55/98 (56.1)	82/145 (56.6)	
Left circumflex artery	25/243 (10.3)	10/98 (10.2)	15/145 (10.3)	
Right coronary artery	81/243 (33.3)	33/98 (33.7)	48/145 (33.1)	
Graft, *n* (%)	0/243 (0)	0/98 (0)	0/145 (0)	
Not determined	0/243 (0)	0/98 (0)	0/145 (0)	
*Number of diseased vessels, n (%)*				0.081
Single	126/243 (51.9)	43/98 (43.9)	83/145 (57.2)	
Double	71/243 (29.2)	31/98 (31.6)	40/145 (27.6)	
Triple	46/243 (18.9)	24/98 (24.5)	22/145 (15.2)	
IABP before PCI, *n* (%)	22/243 (9.1)	11/98 (11.2)	11/145 (7.6)	0.33
VA-ECMO before PCI, *n* (%)	2/243 (0.8)	0/98 (0)	2/145 (1.4)	0.52

*PCI* percutaneous coronary intervention, *AMI* acute myocardial infarction, *IABP* intra-aortic balloon pumping, *VA-ECMO* venoarterial-extracorporeal membrane oxygenation

**Fig. 2:**
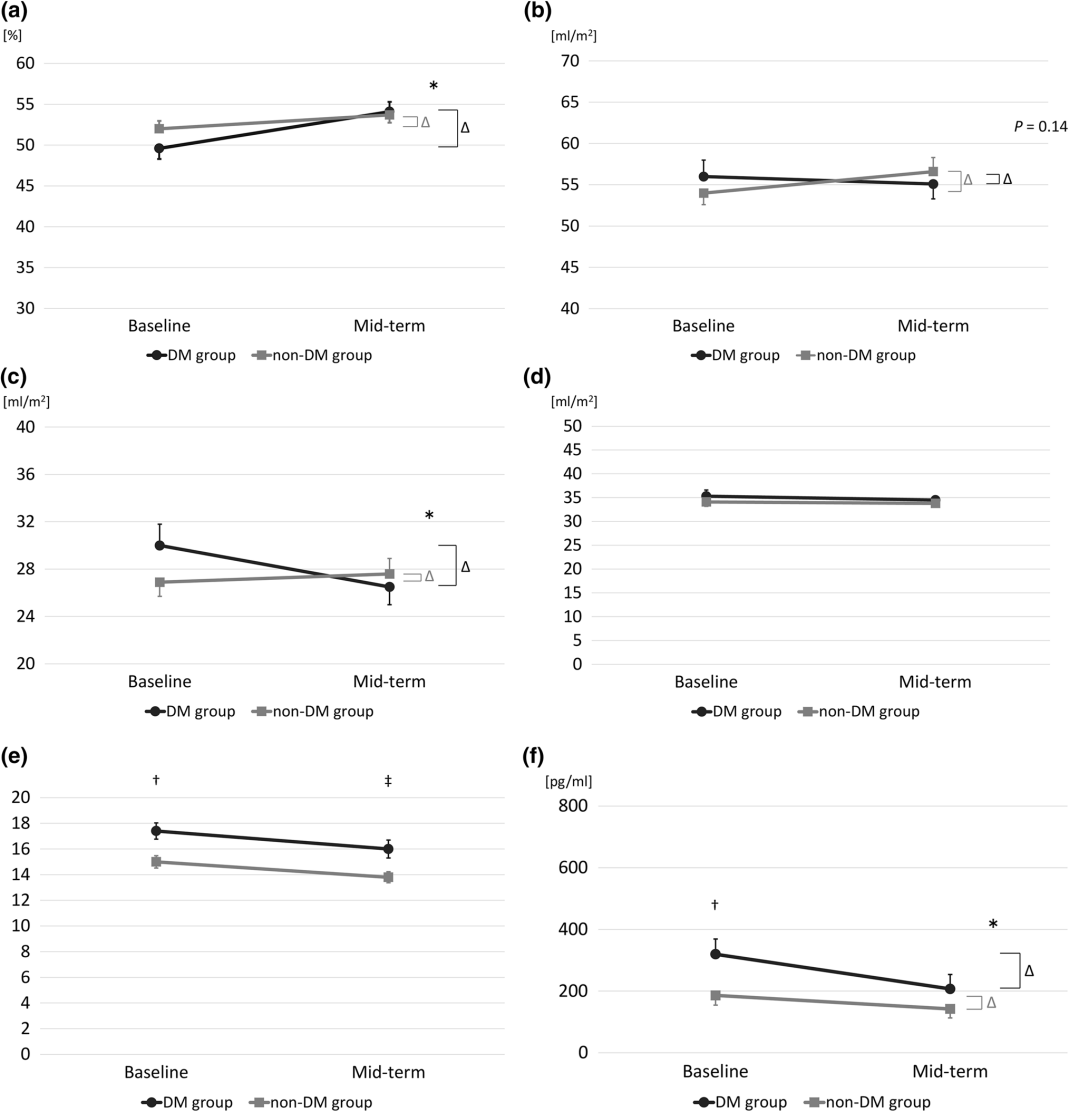
Comparison of temporal changes in left ventricular ejection fraction (LVEF) (**a**), left ventricular end-diastolic volume index (LVEDVI) (**b**), left ventricular end-systolic volume index (LVESVI) (**c**), left atrial volume index (LAVI) (**d**), *E*/*e*′ (**e**), and brain natriuretic peptide (BNP) levels (**f**) from baseline to mid-term follow-up in the patients with diabetes (solid circles) and without diabetes (solid squares). *P < 0.05 changes from baseline to mid-tern follow-up between the 2 groups; ^†^*P* < 0.05 between the 2 groups at baseline; ^‡^*P* < 0.05 between the 2 groups at mid-term follow-up. Data are mean ± SE

Table 3 shows a comparison of the echocardiographic findings and BNP levels at baseline and mid-term follow-up between the 2 groups. The temporal changes in LVEF from baseline to mid-term follow-up were significantly increased in the DM group (4.4% ± 9.9%) compared with the non-DM group (1.7% ± 9.6%; *P* = 0.028). The temporal changes in LVDd, LVDs, RWT, LVESVI, and BNP levels from baseline to mid-term follow-up were significantly decreased in the DM group compared with the non-DM group. The temporal changes in LVEF, LVEDVI, LVESVI, LAVI, *E*/*e*′, and BNP levels from baseline to mid-term follow-up are shown in Fig. 2. *E*/*e*′ was significantly greater in the DM group than in the non-DM group at baseline and mid-term follow-up (17.4 ± 6.4 vs. 15.0 ± 6.0, *P* = 0.001 and 16.0 ± 6.6 vs. 13.8 ± 5.3, *P* = 0.001, respectively). The BNP levels at baseline were significantly higher in the DM group than in the non-DM group (319.6 ± 486.5 vs. 186.1 ± 385.8 pg/ml, *P* = 0.005). The temporal changes in LVEDVI, LAVI and *E*/*e*′ from baseline to mid-term follow-up showed no significant difference between the 2 groups.

**Table 3:** Echocardiographic features and BNP levels at baseline and mid-term in the DM and the non-DM groups

	DM group (*n* = 98)		Non-DM group (*n* = 145)		*P* value, comparison of temporal changes from baseline to mid-tern follow up between the two groups
	Baseline	Mid-term	Baseline	Mid-term	
LVEF, % (*n*, %)	49.6 ± 13.1 (98, 100)	54.1 ± 11.9 (98, 100)	52.0 ± 11.6 (145, 100)	53.7 ± 11.4 (145, 100)	0.028
E/A, (*n*, %)	1.1 ± 0.46 (93, 94.9)	0.96 ± 0.43 (93, 94.9)	1.2 ± 0.69 (140, 96.6)	1.0 ± 0.65 (142, 97.9)	0.32
E/e’, (*n*, %)	17.4 ± 6.4 (95, 96.9)	16.0 ± 6.6 (95, 96.9)	15.0 ± 6.0 (145, 100)	13.8 ± 5.3 (145, 100)	0.57
e’, cm/s (*n*, %)	5.2 ± 1.8 (96, 98.0)	5.0 ± 1.6 (97, 99.0)	5.6 ± 1.7 (145, 100)	5.3 ± 1.7 (145, 100)	0.77
DcT, msec (*n*, %)	192.0 ± 41.1 (96, 98.0)	224.1 ± 54.0 (94, 95.9)	187.1 ± 45.3 (145, 100)	223.0 ± 54.8 (145, 100)	0.51
LVDd, mm (*n*, %)	52.2 ± 7.1 (98, 100)	50.6 ± 6.2 (98, 100)	50.0 ± 6.5 (145, 100)	50.6 ± 6.0 (145, 100)	0.012
LVDs, mm (*n*, %)	32.3 ± 8.4 (98, 100)	35.1 ± 7.7 (98, 100)	34.4 ± 6.8 (145, 100)	34.8 ± 7.1 (145, 100)	0.001
IVST, mm (*n*, %)	10.5 ± 1.7 (98, 100)	10.3 ± 1.7 (98, 100)	10.4 ± 1.8 (145, 100)	9.7 ± 1.8 (145, 100)	0.059
PWT, mm (*n*, %)	10.3 ± 1.6 (98, 100)	10.0 ± 1.8 (98, 100)	10.4 ± 1.5 (145, 100)	9.8 ± 1.6 (145, 100)	0.11
LVMI, g/m^2^ (*n*, %)	117.6 ± 27.3 (98, 100)	110.1 ± 26.0 (98, 100)	113.0 ± 25.8 (145, 100)	106.2 ± 22.9 (145, 100)	0.92
LAVI, ml/m^2^ (*n*, %)	35.3 ± 12.5 (84, 85.7)	34.5 ± 13.1 (89, 90.8)	34.1 ± 10.9 (135, 93.1)	33.8 ± 11.6 (132, 91.0)	0.62
RWT, (*n*, %)	0.40 ± 0.082 (98, 100)	0.40 ± 0.093 (98, 100)	0.43 ± 0.15 (145, 100)	0.39 ± 0.083 (145, 100)	0.034
LVEDV, ml (*n*, %)	98.9 ± 37.8 (98, 100)	95.9 ± 33.6 (98, 100)	93.2 ± 30.3 (145, 100)	96.7 ± 35.6 (145, 100)	0.14
LVESV, ml (*n*, %)	52.8 ± 32.6 (98, 100)	46.3 ± 26.9 (98, 100)	46.4 ± 24.2 (145, 100)	47.0 ± 26.4 (145, 100)	0.046
LVEDVI, ml/m^2^ (*n*, %)	56.0 ± 19.8 (98, 100)	55.1 ± 18.2 (98, 100)	54.0 ± 16.3 (145, 100)	56.6 ± 19.3 (145, 100)	0.14
LVESVI, ml/m^2^ (*n*, %)	30.0 ± 17.9 (98, 100)	26.5 ± 15.0 (98, 100)	26.9 ± 13.7 (145, 100)	27.6 ± 15.2 (145, 100)	0.030
BNP, pg/ml (*n*, %)	319.6 ± 486.5 (94, 95.9)	207.0 ± 427.4 (78, 79.6)	186.1 ± 385.8 (142, 97.9)	142.1 ± 330.4 (119, 82.1)	0.002
Log_10_ BNP, pg/ml (*n*, %)	2.0 ± 0.72 (94, 95.9)	1.9 ± 0.59 (78, 79.6)	1.8 ± 0.62 (142, 97.9)	1.8 ± 0.53 (119, 82.1)	0.037

*LVEF* left ventricular ejection fraction, *DcT* deceleration time, *LVDd* left ventricular internal dimension in diastole, *LVDs* left ventricular internal dimension in systole, *IVST* interventricular septum thickness, *PWT* posterior left ventricular wall thickness, *LVMI* left ventricular mass index, *LAVI* left atrial volume index, *RWT* relative wall thickness, *LVEDVI* left ventricular end-diastolic volume index, *LVESVI* left ventricular end-systolic volume index, *BNP* brain natriuretic peptide

**Fig. 3:**
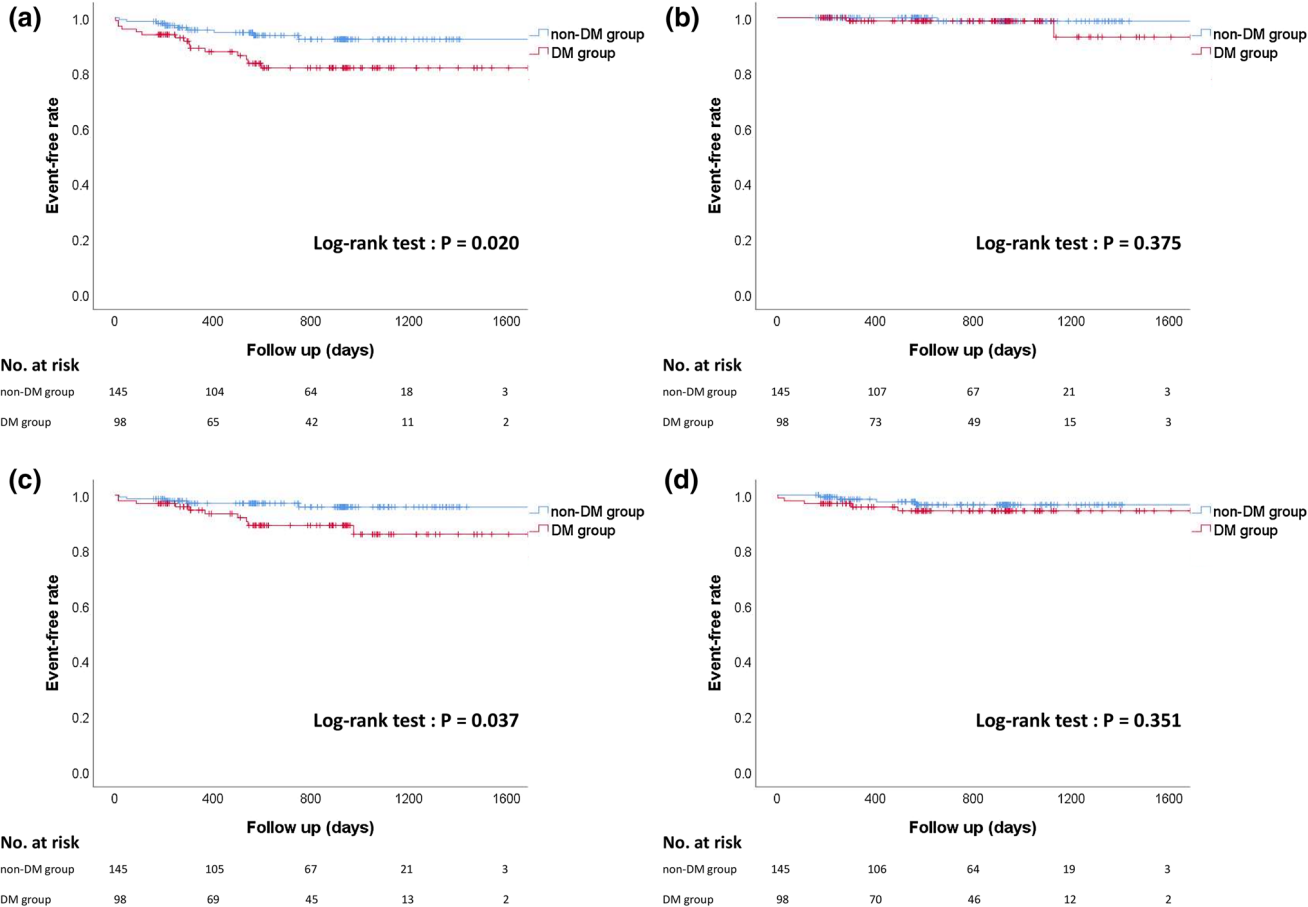
Kaplan–Meier curves for major adverse cardiac events (MACEs) (**a**), all-cause death (**b**), heart failure (HF) hospitalization (**c**), and non-fatal myocardial infarction (MI) (**d**) between the DM and non-DM groups. The *P* values were calculated using a log-rank test

The Kaplan–Meier curves are shown in Fig. 3. During the study period, the number of MACEs was 24, and the number of HF hospitalizations was 15. The incidence of MACEs was significantly higher in the DM group than in the non-DM group (log-rank test; *P* = 0.020). The rate of HF hospitalization was significantly higher in the DM group than in the non-DM group (log-rank test; *P* = 0.037). No significant differences in all-cause death and non-fatal MI were found between the 2 groups. In multivariate Cox regression analyses, DM was significantly associated with MACEs (HR 2.79, 95% CI 1.20–6.47, *P* = 0.017) and HF hospitalization (HR 3.62, 95% CI 1.19–11.02, *P* = 0.023) after controlling age, sex and LVR (Model 1 in Tables 4 and 5). LVR was also significantly associated with MACEs (HR 2.44, 95% CI 1.03–5.78, *P* = 0.044) and HF hospitalization (HR 3.76, 95% CI 1.15–12.32, *P* = 0.029) after controlling age, sex and DM (Model 1 in Tables 4 and 5). However, stress hyperglycemia was not significantly associated with MACE and HF hospitalization after controlling age, sex and LVR (Model 2 in Tables 4 and 5).

**Table 4:** Multivariate Cox regression analysis predicting MACEs

Dependent variable: MACE			
Independent variables	HR	95% CI	*P* value
*Model 1*			
Age (per 1 year)	1.02	0.99–1.06	0.23
Female sex (vs. male sex)	0.69	0.20–2.40	0.55
DM	2.79	1.20–6.47	0.017
LVR	2.44	1.03–5.78	0.044

Dependent variable: MACE			
Independent variables	HR	95% CI	*P* value
*Model 2*			
Age (per 1 year)	1.02	0.99–1.06	0.21
Female sex (vs. male sex)	0.56	0.16–1.94	0.36
Stress hyperglycemia	1.64	0.68–3.95	0.27
LVR	2.09	0.89–4.92	0.09

*MACE* major adverse cardiac event, *HR* hazard ratio, *CI* confidence interval, *DM* diabetes mellitus, *LVR* left ventricular remodeling

**Table 5:** Multivariate Cox regression analysis predicting HF hospitalization

Dependent variable: HF hospitalization			
Independent variables	HR	95% CI	*P* value
*Model 1*			
Age (per 1 year)	1.03	0.99–1.08	0.17
Female sex (vs. male sex)	1.35	0.35–5.23	0.66
DM	3.62	1.19–11.02	0.023
LVR	3.76	1.15–12.32	0.029

Dependent variable: HF hospitalization			
Independent variables	HR	95% CI	*P* value
*Model 2*			
Age (per 1 year)	1.03	0.99–1.08	0.16
Female sex (vs. male sex)	1.01	0.27–3.76	0.98
Stress hyperglycemia	1.82	0.58–5.74	0.31
LVR	3.06	0.96–9.75	0.059

Abbreviations are the same as Table 4

## Discussion

The present study included 243 consecutive patients with STEMI who underwent reperfusion therapy and were categorized into the DM group (*n* = 98) and non-DM group (*n* = 145). The structural changes of the patients’ hearts were examined with echocardiography both at index admission and mid-term follow-up. First, we demonstrated that the DM group had a higher MACEs and HF hospitalization rate than the non-DM group. Second, the structural changes in LV, represented by LVDd, LVDs and LVESVI, were significantly decreased in the DM group compared with the non-DM group. LV functional changes represented by LVEF were significantly increased in the DM group compared with the non-DM group. Third, the degree of temporal decrease in BNP levels from baseline to mid-term follow-up was significantly greater in the DM group than in the non-DM group. Our study suggests that the patients with DM after STEMI have the inverse correlation between MACEs and the LV structural changes. However, we performed multivariate Cox regression analyses predicting MACEs and HF hospitalization, both DM and LVR were significantly associated with MACEs and HF hospitalization. Meanwhile, we performed multivariate Cox regression analyses predicting MACEs and HF hospitalization with the independent variables changed from DM to stress hyperglycemia, stress hyperglycemia was not significantly associated with MACEs and HF hospitalization.

As several studies demonstrated, DM is a strong risk factor of the progression of HF [7, 24–26]. Patients with DM have from 2 to 5 times greater risk of HF than those in the general population [24, 27]. Generally, patients with DM, even those without symptomatic HF, tend to have LV diastolic dysfunction in the presence of increasing LV stiffness and LV mass with normal systolic function [25, 28]. In our study, *E*/*e*′, or the index of diastolic function, was significantly higher in the DM group than in the non-DM group both at baseline and mid-term follow-up, consistent with the results of previous studies; However, LAVI, or an indirect correlate of LV filling pressures over time, was not significantly different between the 2 groups both at baseline and mid-term follow-up. This may be because *E/e’* that includes the movement of mitral annular early diastolic velocity (*e’*) can reflect changes in the myocardial movement more directly than measurement of LAVI [29].

The progression of HF in patients after MI is mainly associated with LVR [1], which is a heterogeneous process affected by various factors, including infarct size, transmural infarction, microvascular obstruction, myocardial hemorrhage, and advanced patient age [30, 31]. One prospective single-center study demonstrated that LVR after successful primary percutaneous transluminal coronary angioplasty (PTCA) occurred despite preservation of regional and global LV functions measured using echocardiography, and the presence of LVR at 6 months after AMI was significantly associated with cardiac death and hospitalization for chronic HF [32]. Meanwhile, in the modern era of primary PCI and OMT, van der Bijl et al. echocardiographically investigated the interaction between LV post-infarct remodeling with LV systolic function and the long-term prognostic impact of such remodeling [22]. During 94 months follow-up, they showed no significant differences in long-term mortality between LV remodelers and non-remodelers, and that the LV remodelers had a significantly higher HF hospitalization rate than the non-remodelers. These 2 studies both demonstrated that LV post-infarct remodeling after successful PCI was associated with HF hospitalization, not with long-term mortality.

By contrast, the present study demonstrated that LVESVI changes from baseline to mid-term follow-up were significantly decreased in the DM group compared with the non-DM group. LVEDVI changes also tended to decrease in the DM group than in the non-DM group. In other words, patients with DM echocardiographically presented reverse remodeling to a larger extent during 6 to 11 months after STEMI than those without DM. Previous studies have demonstrated that infarct size was associated with LVR [30, 33] and anterior MI presented a significantly larger infarct size than non-anterior MI [34]. In the present cohort, the accurate infarct size could not be shown because we did not routinely perform cardiac magnetic resonance imaging (CMR) and single photon emission computed tomography (SPECT) to detect infarct size. Additionally, the rate of infarct-related artery was not significantly different between the 2 groups. The reason why the DM group echocardiographically presented reverse remodeling than the non-DM group is unclear in our study.

Nevertheless, we found that MACEs and HF hospitalization rate were significantly higher in the patients with DM than in those without DM. The echocardiographic substudy in the SAVE trial also reported that the increased incidence of HF after MI in patients with DM was not explained by the increasing trend for LVR in the nondiabetic patients with similar-sized infarcts [35]. The findings that patients with DM after STEMI may develop HF with less LV enlargement than those without DM after STEMI are contrary to the established concepts that LVR progresses to HF in all patients. Furthermore, one multicenter and prospective study demonstrated that DM remains an independent predictor of HF hospitalization in patients with a modern treatment of MI including acute reperfusion therapy followed by optimal medical therapy, although this higher risk is not associated with a decreased LVEF or an increased propensity to LVR but with LV diastolic dysfunction [36]. As mentioned in these 2 studies, this inverse relationship between LVR and HF progression in patients with DM may be associated with increased filling pressure, which is supported by the finding that BNP levels at baseline and mid-term follow-up were higher in the DM group than in the non-DM group. To our knowledge, this is the first study to show that the extent of LVR was less in patients with DM than in those without DM, although BNP levels were higher in the patients with than in those without DM after STEMI. Moreover, a previous study demonstrated that the patients with DM, even with short diabetes duration and good glycemic control, impaired diastolic function [37]. In the present study, the patients with DM after STEMI were significantly increased the rate of MACEs, notably HF hospitalization, despite of less LVR. This suggests that the patients with DM after STEMI may be hospitalized for HF because of increased filling pressure before LVR occurs.

However, according to multivariate Cox regression analyses shown in Tables 4 and 5, the patients with both DM and LVR had worse clinical outcomes including MACEs and HF hospitalization. In brief, if we focused on patients with LVR, those who had DM after STEMI had significantly higher HF hospitalization rate, despite that the patients with DM after STEMI tended to present reverse remodeling. Our data indicate that treatment of patients with DM after STEMI is particularly important for the prevention of LVR, including administration of an angiotensin receptor neprilysin inhibitor [38, 39] and sodium glucose cotransporter type 2 inhibitors [40].

## Study limitations

This study has several limitations. First, because this study was a single-center retrospective observational study with a small number of patients, selection bias may exist. We could not conduct propensity score matching to fit the difference in background between the 2 groups, as the sample size was relatively small. Therefore, the difference between the 2 groups may have affected the results. Second, as we performed the echocardiographic analysis on-site, the echocardiographic measurements may have differed between ultrasonographers. Additionally, as the number of LAVI measurements both at baseline and mid-term follow-up was less than other measurements owing to inappropriate image detection, it may not reflect the exact differences in LV filling pressures between the 2 groups. Third, a beta error is possible in the comparisons between the DM and non-DM groups, as the total study population was relatively small (n = 243). Fourth, we could not evaluate the long-term outcomes because the follow-up duration may not be sufficiently long (median, 621 days [interquartile range, 304–963 days]). A larger sample size and longer follow-up might be needed. Fifth, there may have been some differences in the extent of LVR between cases since mid-term echocardiographic follow-up duration is slightly too long to evaluate LVR. Finally, the peak CK level, that was significantly lower in the DM group than in the non-DM group, could not correctly reflect the infarct size, because the patients with delayed arrival included in the present study. We could not evaluate the accurate infarct size, because we did not routinely perform CMR and SPECT after the admission.

## Conclusions

As compared with the patients without DM, those with DM after STEMI had a higher MACEs and HF hospitalization rate; nevertheless, the extent of LVR was less in the patients with than in those without DM. However, the patients with both DM and LVR after STEMI were significantly associated with worse clinical outcomes including MACEs and HF hospitalization, which suggests that minimizing LV remodeling after STEMI is particularly critical in patients with DM.
